# Development and Optimization of Djulis Sourdough Bread Using Taguchi Grey Relational Analysis

**DOI:** 10.3390/foods9091149

**Published:** 2020-08-20

**Authors:** Pei-Ling Chung, Ean-Tun Liaw, Mohsen Gavahian, Ho-Hsien Chen

**Affiliations:** Department of Food Science, National Pingtung University of Science and Technology, Neipu 91201, Pingtung, Taiwan; plchung@tajen.edu.tw (P.-L.C.); alexliaw@mail.npust.edu.tw (E.-T.L.); mohsengavahian@yahoo.com (M.G.)

**Keywords:** bakery products, bread, djulis, food quality, optimization, product development, Taguchi grey relational analysis, texture profile analysis, sensory attributes, sourdough

## Abstract

Bakery products made from naturally fermented sourdough show a diversified flavor and nutritional profile. Djulis (*Chenopodium formosanum*), known as red quinoa or Taiwan djulis, originally cultivated by Taiwanese indigenous people in mountain areas in eastern and southern Taiwan, has a high nutritional value and characteristic properties. In the present study, a new bakery product (djulis sourdough bread) was developed and a combination of the Taguchi method coupled with grey theory was utilized to optimize the baking parameters (product formulation). Five main factors, i.e., djulis sourdough (A), hulled djulis (B), oil type (C), a mixture of bread flour (wet gluten content of 29.0%) and a high-gluten flour (wet gluten content of 35.5%) (D), and honey (E), (each at four levels) were chosen for the Taguchi experiment design (L_16_(4)^5^). Dependent parameters were the data from texture profile analysis (brittleness, springiness, cohesiveness, gumminess, and chewiness), color analysis (L*, a*, and b*), and sensory evaluation (appearance, aroma, bitterness, sourness, chewiness, and overall acceptance) of the final product. Taguchi grey relational analysis successfully determined the optimal conditions based on combined parameters (5 factors), which highlighted the advantages of this innovative optimization technique. The result shows that the optimal formula for producing a djulis sourdough bread with the best texture, color, and sensory qualities was A3B1C1D2E2, i.e., 20% djulis sourdough, 0% addition of hulled djulis, 8% unsalted butter, 80% wheat flour + 20% high-gluten flour, and 10% honey, respectively. Such a novel application could be a reference for improving the quality of bakery products in the industry. Moreover, it seems that the new bakery product developed in this study has good potential to be commercially produced after further nutritional and economic analysis.

## 1. Introduction

Djulis (*Chenopodium formosanum* Koidz.) belongs to the Amaranthaceae family and *Chenopodium* genus. According to Encyclopedia Britannica, the Amaranthaceae family includes about 175 genera and more than 2500 species. Many species, including beets and quinoa, are considered staple food crops, and some are cultivated as garden ornamental plants [1]. Among them, amaranth species are located mainly in tropical and subtropical areas. Grain amaranth yields tiny seeds that can be used as a grain to make flour, porridge, and other foods. For instance, amaranth grain can be processed to be added into several products including baby food, cakes, and cookies. In addition, amaranth grain has a high concentration of lysine, that is, an essential amino acid for the biosynthesis of proteins, which is vital for human tissue development and healing. Furthermore, this grain is rich in calcium, phosphorus, iron, potassium, zinc, vitamin E, and vitamin B-complex [2]. Previous studies have shown bioactive effects for species of the Amaranthaceae family [3]. For example, Sánchez-Urdaneta et al. fed rats with breads made with amaranth (*Amaranthus dubius* Mart. ex Thell) flour and observed that consumption of amaranth-enriched bread enhanced lipid profiles of rats and prevented metabolic and cardiovascular diseases due to its hypoglycemic and hypolipidemic effects [4]. Moreover, an in vivo study revealed that phenylpropanoid extract from *Halosarcia indica* (Willd.) has analgesic and anti-inflammatory effects on Wistar albino rats [5].

In Taiwan, djulis is also called Taiwan djulis or Taiwan red quinoa and has different strains with diverse colors. This plant has been cultivated mainly in eastern and southern areas of Taiwan (Taitung and Pingtung) as a cereal crop and was previously used for worship purposes and decorations in seasonal festivals by Taiwanese indigenous people (Taiwan aborigines). In recent years, physicochemical and bioactive characteristics as well as preventive healthcare applications of this crop get attention [6]. For instance, Hong et al. demonstrated that djulis extract could protect skin from UV-induced damage [7]. Additionally, Lee et al. found that the early stages of chemically induced colon carcinogenesis were suppressed in mice after feeding them djulis for 10 weeks [8]. However, there is limited information about the possibility of using this crop for developing a bakery product with optimal characteristics.

The Taguchi method is a systematic approach for experimental design and analysis. Recently, this approach has gained popularity to be used in various sectors of the industry for new product development and quality improvement in an economical way. Previous work demonstrated that the Taguchi method was used for the optimization of the conditions for submerged culture at a laboratory-scale study and resulted in the development of an upscaled fermentation process that could yield a high concentration of monacolin K [9]. Although the application of other optimization approaches (e.g., response surface methodology) has been widely explored for food processing [10,11], there are only limited studies in the literature that explored the applicability of the Taguchi method for developing new bakery products. Moreover, it seems that the limitations of other optimization approaches (e.g., response surface methodology; RSM) can be addressed by the application of the Taguchi method. For example, A study conducted by Chen et al. showed the applicability of the Taguchi technique for the quality improvement of egg–shortening cakes [12]. Basically, when applying the Taguchi method in process optimization, the optimum combination is determined based on one quality characteristic at a time. However, in practice, in production lines usually the process involves more than one characteristic (multiple objectives). Consequently, the effects of non-linear interactions between control factors exist and cannot be ignored. Therefore, the grey system theory is an approach that can be employed to optimize multi-characteristic processes.

The theory of the grey system was proposed by Professor Deng Julong in the 1980s [13]. Grey relational analysis (GRA), procured from grey system theory, is a measurement technique to determine the relationship between sequences through the analysis of a limited number of data [14,15]. The relational grade is defined as measuring the relevance between or two sequences or two systems and can be used to describe the trend relationship between a reference sequence (objective or ideal sequence) and a comparative sequence in a specific system. In such studies, a relational grade approaching 1 suggests that the reference sequence and the comparative sequence tend toward concordance. On the other hand, a relational grade approaching 0 indicates that the reference sequence and the comparative sequence do not tend toward concordance completely. This technique requires small quantities of data, and the data are not restricted to specific statistical distributions, which makes GRA superior to classical statistical methods. In previous studies, Chen et al. used GRA successfully to investigate the adulterated cases of commercial soybean sauces [16]; Chen et al. employed GRA effectively to identify and classify undried roselle samples frozen at −20 °C, and roselle samples dried correctly at 20, 50, 75, and 85 °C, respectively [17]. Moreover, associating the Taguchi method with GRA has also shown a powerful tool to optimize the multiple performance characteristics in the food manufacturing process. Chen et al. applied the Taguchi grey relational analysis method to optimize the fish drying process based on performance characteristics such as color measurement value (L*, a*, b*), thiobarbituric acid value (TBA), and shear stress value [18]. Chung et al. used a grey-based Taguchi approach to improve beneficial monacolin K, *Monascus* pigment synthesis, and to decrease the adverse metabolite, citrinin, in the fermentation of *Monascus purpureus* [19].

Despite the progress in the application of grey system theory in several fields, this application of this innovative approach is an ongoing topic in the food industry. For example, the grey system theory has not been well-explored for optimization of products such as sourdough breads, which are believed to have improved shelf life and sensory properties such as flavor, aroma, and texture (mainly due to fermentation by yeasts, *Aspergillus*, and lactic acid bacteria) [20,21]. Specifically, developing a new djulis sourdough-based bread need a tremendous optimization that has not been explored in the literature. Therefore, this study aims to develop a new naturally fermented bread product (djulis sourdough bread) with acceptable palatability by utilizing the Taguchi–GRA method as an innovative optimization approach that can serve as a potential practice for industrial baking. In this regard, such a novel approach was utilized through an overall evaluation process for the optimization of djulis sourdough bread manufacturing based on 14 characteristics (objectives) including attributes obtained by texture profile analysis (TPA), measurement of L*, a*, and b* values by a colorimeter, and sensory evaluation.

## 2. Materials and Methods

### 2.1. Questionnaire and Experimental Strategy

Thirty experienced bakers were invited to reexamine the standard formula used for making general commercial round-top white bread set up by the China Grain Products Research and Development Institute (New Taipei City, Taiwan) [22]. This standard formula is as follows (ratio of materials): 100% high-gluten flour (HGF), 10% fine granulated sugar, 8% butter, 4% fresh yeast, 54% water, 12% egg, 2% salt, and 4% milk powder. Based on the questionnaire responses, feedback, and discussions collected from these 30 experienced bakers, five potential influential factors that would affect bakery product quality were selected and identified for the experimental design and were then used to develop the djulis sourdough bread. These five factors were the addition/non-addition of djulis sourdough, honey, wheat flour (WF) or HGF, addition/non-addition of hulled djulis, and butter/oil. The abovementioned evaluations were performed to design an experiment with a specified number of tests as well as specified ranges for each test.

### 2.2. Experimental Preparation

Before running the experiment, a bunch of organic djulis was added and mixed with leftover baguette dough for four hours to form a djulis sourdough. Then the djulis sourdough was cultured in a refrigerator at 4–7 °C. Re-culturing was performed every seven days. In this regard, an appropriate amount of djulis sourdough was added into flour and water, kept at room temperature for three hours, and then refrigerated for the continuation of the low-temperature fermentation process.

**Figure 1:**
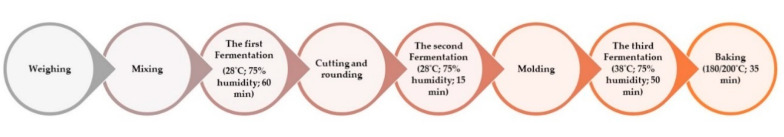
A flow chart representing djulis sourdough bread preparation in the present study.

The preparation of djulis sourdough bread was based on formulas derived from the factor/level assignments. Three fermentation processes were conducted for djulis sourdough preparation, i.e., the first fermentation was run at 28 °C, 75% of humidity for 60 min; after cutting and rounding, the second fermentation was run again at 28 °C, 75% of humidity for 15 min. Then after the appearance shaping, 38 °C, 75% of humidity for 50 min were applied in the third fermentation. After fermentation, the sourdough was baked for 35 min at 180–200 °C (top and bottom heat). The flow chart for making the djulis sourdough bread is shown in Figure 1.

### 2.3. Materials

Djulis was collected from Machia Township (Pingtung County, Taiwan). A WF sample with a wet gluten content of 29.0%, protein content of 10.0%, and ash content of 0.6% and an HGF with a wet gluten content of 35.5%, protein content of 12.5%, and ash content of 0.4% were obtained from Yuan Shan Food Co., Ltd. (Pingtung, Taiwan); Anchor unsalted butter, originally from New Zealand, obtained from Tehmag Foods Co. (New Taipei City, Taiwan); camellia oil (Yuan Shan Food Co., Ltd., Pingtung, Taiwan); Italian olive oil obtained from Tehmag Foods Co. (New Taipei City, Taiwan); lard (I-MEI Foods Co., Ltd., Pingtung, Taiwan); honey (Longan honey, The Chen’s Honey, Pingtung, Taiwan); eggs (PX Mart, Pingtung, Taiwan); Anchor full cream milk powder, originally from New Zealand, obtained from Yu Hsuan Inc. (Pingtung, Taiwan); salt (Yu Hsuan Inc., Pingtung, Taiwan); charcoal-filtered water (PX Mart, Pingtung, Taiwan); and yeast (Yu Hsuan Inc., Pingtung, Taiwan).

### 2.4. Sensory Evaluation and Instrumental Measurement

#### 2.4.1. Sensory Evaluation Analysis

The seven-point hedonic scale was adopted to assess the overall round-top djulis sourdough bread acceptability. Such a test was performed by 60 participants (between 20 and 35 years old) who were chosen randomly from students of the National Pingtung University of Science and Technology (NPUST). Appropriate guidance and training for all panelists were provided before conducting the sensory evaluation. Four categories of sensory attributes were stated on the score sheet as follows: appearance, smell/taste (aroma, bitterness, and sourness), texture (chewiness), and overall acceptability. Each item was scored between 1 and 7 (1: dislike extremely, 2: dislike moderately, 3: dislike slightly, 4: neither like nor dislike, 5: like slightly, 6: like moderately, 7: like extremely). Items could not be scored more than once. There were a total number of 16 slices of bread that needed to be practiced by a panelist per day. After the evaluation had been completed for one slice of bread, a 15 s interval was required before the next practice. During each evaluation, the external appearance of the sliced bread was first observed and scored. Afterward, the scoring was performed one by one for aroma, bitterness, sourness, chewiness, and overall acceptability. The sensory evaluation tests were performed in triplicate (*n* = 3) on three days, meaning that each member of the sensory evaluation panel (60 members) need to repeat the tests three times on different days. The final score of each item was calculated and obtained by averaging the three-replicate data.

#### 2.4.2. Texture Profile Analysis

Oven-fresh round-top djulis loaves were subjected to TPA using a texture analyzer (TA-XT-Plus, Stable Micro Systems, Ltd., Godalming, UK) based on a standard method according to approved methods of the American Association of Cereal Chemists (AACC), as described by Amigo et al. [23]. First, each sample was sliced into 1.25-cm-thick slices, the slices at both ends were discarded, and the slices from the middle portion of each sample were used for analysis. During the testing process, two bread slices were stacked and analyzed using a 36-mm-diameter probe, with a compression ratio of 25% and a probe compression speed of 5 m/s. Each type of sample was compressed four times using eight slices from the middle portion, and data related to brittleness, springiness, cohesiveness, gumminess, and chewiness were recorded and calculated using the developed software (TA-XT-Plus, Stable Micro Systems, Ltd., Godalming, UK) [24,25].

#### 2.4.3. Colorimeter Analysis

After cooling for one hour, round-top djulis loaves were sliced into 1.25-cm-thick slices, and slices from the middle portion of each sample were subjected to color and lightness measurements using a colorimeter (Minolta CR 310, Konica Minolta Sensing Singapore Pte. Ltd., Jurong East, Singapore). Color and lightness values were expressed as L*, a*, and b*, with L* representing lightness (L* for brightest white = 100) or darkness (L* for darkest black = 0); a* representing the red/green component (+a*: red, −a*: green); and b* representing the yellow/blue component (+b*: yellow, −b*: blue). The average value of six measurements was used for each parameter [26].

### 2.5. Data Analysis Models

Analyses of multiple quality characteristics of the baked djulis sourdough bread samples were performed using the novel combination approach, the Taguchi–GRA method, as shown in the following.

#### 2.5.1. Taguchi Method

With the Taguchi method [27], an orthogonal array is first constructed by assigning known or assumed control factors and noise factors. Accordingly, the optimal parameter levels are determined with the minimum number of experiments. The orthogonal array is denoted by L_n_(X^m^), where n is the number of columns of the array (i.e., the number of parameter and level combinations in the experiment), X is the number of levels, and m is the number of rows of the array (i.e., the number of factors). The orthogonal array used in the present study is denoted by L_16_(4^5^), meaning that five control factors with four levels were used in 16 bakery product experiments. The five control factors used in this study were djulis sourdough (A), hulled djulis (B), butter/oil (C), Taiwan flour (D), and honey (E). Four levels (the ratio of formula) were set for each control factor (Table 1) in which the common ingredients were 3.5% fresh yeast, 54% distilled water, 12% egg, 2% salt, and 4% milk powder. The selected orthogonal array L_16_(4^5^) and factor/level assignments are shown in Table 2 as the mean of three replicates.

#### 2.5.2. Calculation of S/N (Signal-to-Noise Ratio) Values

Experimental data of those multiple quality characteristics in the orthogonal table were used to calculate the signal-to-noise ratio (S/N ratio, η). The S/N ratio did create a transformation function of the repetition data to another value and was used as a measure of the variation present in the experiment. S/N is a function indicator that measures performance, with higher S/N values indicating smaller quality losses. There are three types of quality characteristics for S/N values: nominal-the-best, smaller-the-best, and larger-the-best. In this study, we aimed to find the optimal operational parameters for the manufacturing of djulis sourdough bread retaining taste, nutrients, and supple flavors. Accordingly, the larger-the-best loss function was, therefore, used to calculate the S/N ratio as described in Equation (1).

(1) $$\eta \;=\;-10\;\log(\frac{1}{n}\sum_{i=0}^{n}1/y_{i}^{2})$$

where *y_i_* is the *i*th value of the quality attribute, and *n* is the number of trials.

#### 2.5.3. Algorithm of GRA

GRA was used to develop multiple quality characteristics of the djulis sourdough bread, to assess the optimal combination of parameters that best satisfies all the quality characteristics and to proceed with overall evaluation. These quality characteristics (dependent parameters) include color values, sensory attributes, and textural property. Data pretreatment was performed before employing GRA for data normalization, i.e., normalizing the raw data or their S/N ratios in the range of 0–1. All these characteristics and their S/N ratios were in the nature of the larger-the-better characteristics. 

According to the literature [12,13,16,17], normalized functions could firstly be represented as Equation (2).

(2) $$X_{i}^{*}\left(k\right)=\frac{X_{i}\left(k\right)-\min[X_{i}\left(k\right)]}{\max[X_{i}\left(k\right)]-\min[X_{i}\left(k\right)]}$$

where $X_{i}^{*}\left(k\right)$ is normalized raw data, $X_{i}^{*}\left(k\right)$ is a comparative sequence with *k*th entities, *i* = 1, …, *m*; *k* = 1, …, *n*; and $\max[X_{i}\left(k\right)]$ and $\max[X_{i}\left(k\right)]$ are the maximum and minimum ones in the comparative sequence.

The grey relational grade (GRG) could depict the degree of relationship between a reference sequences (objective sequence or ideal sequence) and a comparative sequence in which it is comprised of 14 characteristics including attributes obtained by TPA; measurement of L*, a*, and b* values; and sensory evaluation. Equations (3)–(5) were used for the calculations related to GRA.

Let *X*_0_* (k)* be the reference sequence with *k*th entities, that is,

*X*_0_*(k)* = *{x*_0_ (1), *x*_0_ (2), …, *x*_0_*(n)}*, (3)

where *k* = 1, 2, 3, …, *n*.

Let X*j (k) be the compared sequence; each X*_j_ possess the same number of entities as *X*_0_, that is,

X*_j_(k) = {x*_j_ (1), x*_j_ (2), …,x*_j_(n)}, (4)

where *k* = 1, 2, 3, …, *n*.

The grey relational coefficient between the reference sequence of *X*_0_ and the compared sequence X*_j_ and at the *k*th entity are described in Equation (5):

(5) $$\gamma \left({Χ}_{0}\left(k\right),{Χ}^{*}{}_{j}\left(k\right)\right)=\frac{\Delta \min+\xi \Delta \max}{\Delta_{0j}\left(k\right)+\xi \Delta \max}$$

where

- $\Delta_{0j}\left(k\right)$ is the absolute difference value between *X*_0_ and X*_j_ at the *k*th entity, that is, $\Delta_{0j\;}\left(k\right)=\left|x_{0}\left(k\right)-{x^{*}}_{j}\left(k\right)\right|$,
- $\Delta \max=\overset{\max}{\forall j}\;\overset{\max}{\forall k}\Delta_{0j}\;\left(k\right)$,
- $\Delta \min=\overset{\min}{\forall j}\;\overset{\min}{\forall k}\Delta_{0j}\;\left(k\right)$,
- ξ∈[0,1] is the distinguishing parameter in controlling the resolution between Δmax and Δmin. For this case, the value of 0.5 was selected.

The optimal settings of process parameters combine multiple quality characteristics into one integrated numerical value, that is, GRG. This parameter for the sequence of X*_j_ is represented in Equation (6).

(6) $$\Gamma_{0j}=\Gamma \left({Χ}_{0},{Χ}_{j}\right)=\overset{n}{\underset{k=1}{\sum }}w_{k}\gamma \;\left({Χ}_{0}\left(k\right),{Χ}_{j}\left(k\right)\right)$$

where *W_k_* is the *k*th weighting of $\gamma_{0j}$.

The value of the GRG (Γ*_*0*j_* in Equation (6)) represents the level of similarity between the comparative sequence X*_j_ (the *j*th of the experimental trials) and the referential sequence *X*_0_. The GRG of each experimental trial can be treated as a response (Γ*_j_*) for each row of the orthogonal array of Table 2. The response graph can be set up by grouping the response values of the corresponding same factor levels of the column in the array, taking the sum, and dividing by the number of responses, as follows:

(7) $$L_{j}=\frac{\sum_{j=1}^{n}\Gamma_{j}}{n}$$

where Γ*_j_* is the response value of corresponding same factor levels of the column in the array, *L_j_* is the mean response of the corresponding factor level.

### 2.6. Statistical Analysis

All the experiments were analyzed in triplicate. The analysis of variance (one-way ANOVA) was applied to the date to determine the significance of influences of control factors used for making djulis sourdough bread and was performed using an SPSS Statistics V.22.0 for Windows Statistical package (IBM Corporation, Armonk, NY, USA). The differences were significant statistically when *p* < 0.05 using the Duncan multiple range tests.

## 3. Results and Discussion

### 3.1. Appearance and Bread Volume

**Figure 2:**
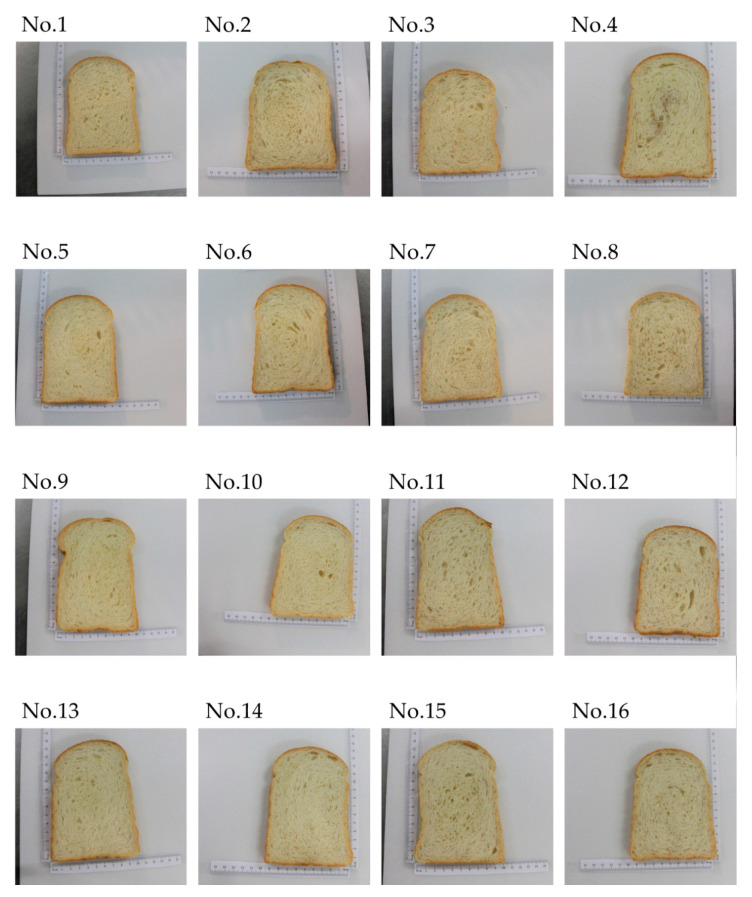
Sixteen numbered round-top djulis bread sliced samples (No. 1 to No. 16) made of different formulas as described in Table 2. No.: number.

Sixteen round-top djulis sliced bread samples were prepared using different formulas based on the L_16_(4^5^) orthogonal array and factor/level assignments (Table 1 and Table 2). These samples were numbered as samples No. 1–16 as can be seen in Figure 2. The first four types of bread in Table 2 (samples No. 1–4 in Figure 2) were prepared without the addition of djulis sourdough. The average length, width, and height of these bread were 30.3, 10.3, and 13.1 cm, respectively. The other 12 types of samples that were prepared with the addition of djulis sourdough (samples No. 5–16 in Figure 2) had smaller sizes, that is, the average length, width, and height of these sourdough breads were 29.9, 10.0, and 11.88 cm, respectively. These observations suggest that the incorporation of djulis in the sourdough bread may reduce the loaf rising. Similarly, previous studies showed that change in the formulation may affect the loaf volume [28,29]. Such changes could be related to several parameters including the effect of the formulation on the gluten network as well as on the fermentation process.

### 3.2. Sensory Evaluation, Texture, and Color Analysis

The raw sensory data from questionnaires (Supplementary Materials; Figure S1) were used to calculate the results of the sensory evaluation of the 16 sliced bread samples (Table 3). According to the results, the trial No. 9 (20% djulis sourdough, 0% addition of hulled djulis, 8% lard, 40% WF + 60% HGF, and 10% honey) consistently gained higher scores on sensory attributes, including appearance, aroma, bitterness, sourness chewiness, and overall acceptance (Table 3). As djulis would release a bitter taste, the sensory evaluation of trial No. 9 showed the highest score on bitterness (less bitter, in the level of like slightly). In terms of sourness, sample No. 9 got the highest score, which showed that incorporation of djulis in the formulation can affect the sourness of the bread. This could be related to both the direct effect of djulis on the final taste of the product as well as its effect on the fermentation process and fermentation products. Similarly, for the appearance the bread, the sensory evaluation team preferred the appearance of sample No. 9. As can be seen in Figure 2, this sample has a unique distribution of air bubbles and color, which is related to the different formulation compared to other samples. Additionally, the panelists found sample No. 4 more chewy, which is in line with the observation about the air bubble distribution (Figure 2) as well as the reduced volume of the sample. This observation depicted that the sensory panelists in this study did not like bitterness in djulis sourdough sliced bread. Nevertheless, it could not be concluded that trial No. 9 was the best product among all bread samples only based on these sensory observations. Objective criterion data from instrumental analyses such as TPA and colorimeter analysis are also equally crucial parameters that should be included in the calculation (Table 3). In other words, an overall evaluation, based on both sensory and instrumental data, is necessary to determine the best set among others. In such cases, different results might be generated depending on the objectives of interest. According to the instrumental data, the addition of djulis affected the color values and textural properties of the final product. It was observed that the addition of djulis can alter the bread color values. Moreover, the instrumental texture analysis data were in line with those of the sensory evaluation. For example, similar to the panelists, TPA also confirmed that sample No. 9 is among the chewiest samples. The results of the present study were in line with those reported in the literature. For example, researchers observed a notable impact of formulation on the textural attributes of bread [28,29].

### 3.3. Calculation of S/N Values of Quality Characteristics

Table 3 shows data on the 14 quality characteristics obtained from sensory evaluation, texture analysis, and colorimetric analysis. Using the Taguchi method, the values of quality characteristics were transformed into S/N values (Table 4), which were then used to determine a formulation with the best quality and lowest variance. When the Taguchi method is used for process optimization, in some cases, a single quality characteristic is set as the target. As a result, experimental results can be shown as a simple linear relationship through the calculation of S/N values, and the best experimental combination can be directly determined from the response graph of the Taguchi orthogonal array. However, in practical production lines of the bakery industry, the investigation of a single quality objective is extremely rare. It means that, for food processing and product development, a number of parameters affect the overall quality of the product. Therefore, process optimization based on a single quality parameter usually cannot provide practical information for the industry. In the present study, 16 experimental trials with multiple quality objectives had to be investigated at a time. As the different quality characteristics had different units and attributes, data incomparability existed in the sequences. Therefore, data preprocessing was required to convert the data of the sequence with distinct scale and dimension into ones having a consistent unified scale and no dimension. The optimization practice in this study has successfully taken into account various processing and quality parameters. Therefore, the grey relational analysis could be employed with available comparable sequences [17].

### 3.4. Grey Relational Analysis

Before using GRA, the S/N values of the various quality characteristics were preprocessed and converted into normalized values ranging from 0 to 1 through Equation (2), as shown in Table 5. The normalized data possessed good consistency and satisfied the three basic conditions for sequence comparison mentioned in the previous section. As the larger-the-best S/N values were calculated for the multiple quality characteristics of the present study, the value of the reference sequence for GRA was set to 1. Therefore, the normalized GRG closer to 1 in the sequence indicated greater closeness to the target value.

With the calculation of GRG, the distinguishing coefficient ζ is set within the range of zero to one (0 < ζ ≤ 1). This ensures that the maximum sequence difference Δ_max_ does not become excessively large and causes a loss of the influencing power of the minimum sequence difference Δ_min_. Although excessively high or low values of ζ will lead to linear biases in data [13], the main function of ζ is to adjust the degree of contrast between the background value and the object being tested. Therefore, the value can be adjusted based on actual needs, as changes in the value of ζ only lead to changes in relative values without affecting the order of the GRG [13]. In the present investigation, a value of 0.5 was used for ζ.

**Figure 3:**
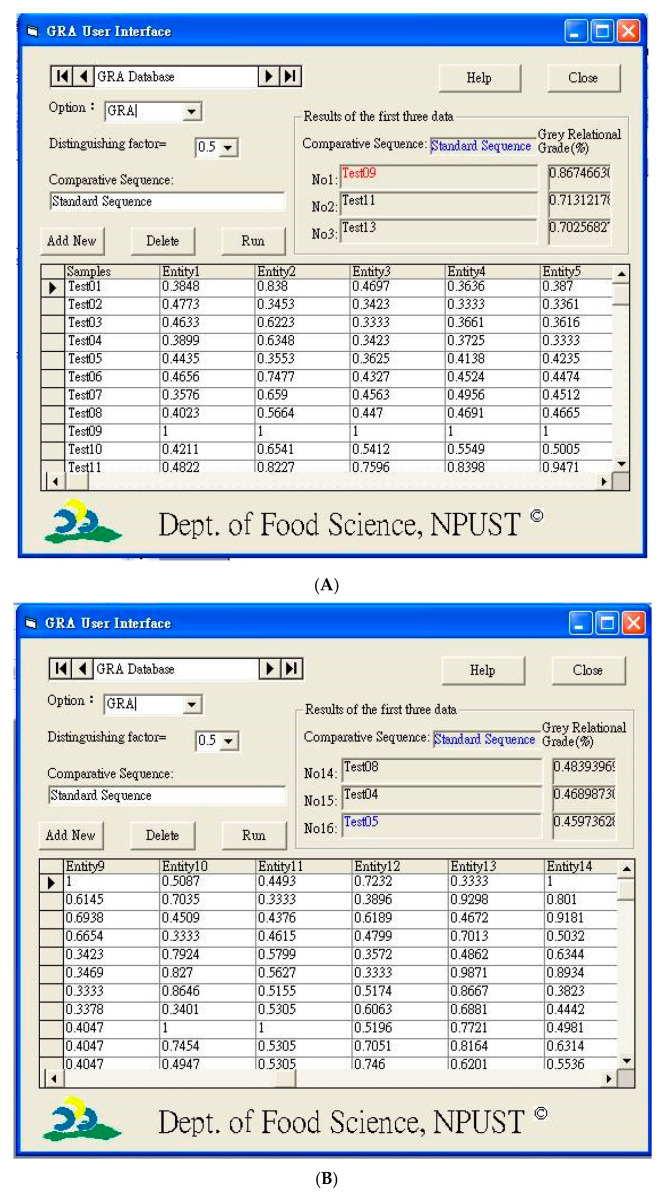
(**A**) First three results obtained directly from the GRA user interface; (**B**) last three results obtained directly from the GRA user interface.

Weightings were assigned to the quality characteristics. Fourteen quality characteristics, including appearance, smell/taste (aroma, bitterness, and sourness), texture (chewiness), overall acceptance, TPA attributes (gumminess, chewiness, brittleness, springiness, and cohesiveness), and colorimetric values (*L*, a*, and b*), were classified into four categories based on the characteristics preferred by consumers who purchase bakery products, and a weighting of 1/4 was assigned to each category (Table 6). The reference sequence (ideal values) was chosen as *X*_0_* (k)* = (1, 1, 1, 1, 1, 1, 1, 1, 1, 1, 1, 1, 1, 1), in which all 14 characteristics employed the concept of the larger-the-better for this work. All 16 sequences were treated as comparative sequences, and each sequence was composed of 14 characteristics (entities). The GRGs of 16 comparative sequence, which were calculated according to Equations (2) and (6), are presented in the last column of Table 6. Then, the grey relational analysis user interface was developed accordingly, to perform computer computing instead of manual calculation (Figure 3).

### 3.5. Parameter Optimizaion

#### 3.5.1. Optimal Factors and Levels

The larger the GRG, the closer the product quality to the objective value. For instance, in Table 6, experimental trial No. 9 seems to be acceptable and closer to the reference sequence (ideal sequence), in which the highest GRG, 0.8675, was obtained. Accordingly, the order of trials (No. 1–No. 16) was rearranged as No. 9 (0.8675) > No. 11 (0.7131) > No. 13 (0.7026) > No. 1 (0.6163) > No. 16 (0.6016) > No. 10 (0.5861) > No. 14 (0.5855) > No. 6 (0.5710) > No. 2 (0.5439) > No. 3 (0.5354) > No. 11 (0.5290) > No. 15 (0.4930) > No. 12 (0.4881) > No. 8 (0.4839) > No. 4 (0.4690) > No. 5 (0.4597). However, non-linear interrelations were observed between the processing parameters in bakery manufacturing. A previous investigation showed that the mean GRG of each experimental trial could be regarded as the response and processed to determine optimal combinations of process parameter levels when a system with multiple performance characteristics is evaluated [18].

**Figure 4:**
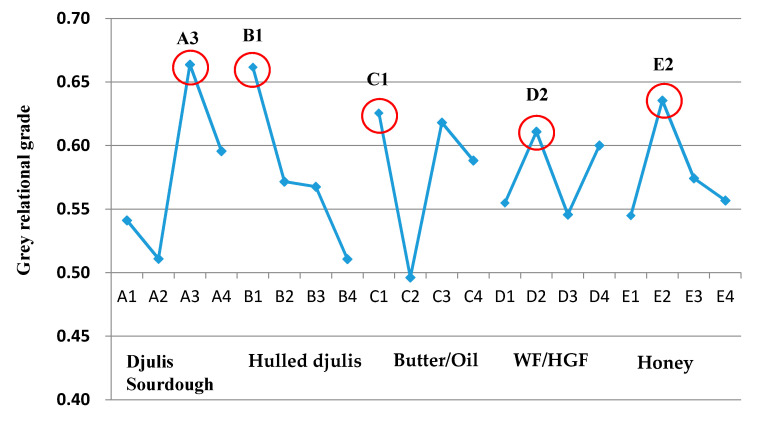
Response graph of grey relational grades of djulis sourdough bread. WF: wheat flour; HGF: high-gluten flour.

Based on the L_16_(4^5^) orthogonal array (Table 2), the GRG for all 16 experimental trials (Table 6), and Equation (7), mean responses of the control level were calculated by taking the sum of the same levels in the column divided by the number of levels. For instance, values of A1, A2, A3, and A4 were 0.541 ((0.6163 + 0.5439 + 0.5354 + 0.4690)/4), 0.511 ((0.4597 + 0.5710 + 0.5290 + 0.4839)/4), 0.664 ((0.8675 + 0.5861 + 0.7131 + 0.4881)/4), and 0.596 ((0.7026 + 0.5855 + 0.4930 + 0.6016)/4), respectively. Likewise, mean responses were calculated for all factor levels of B, C, D, and E to generate a response graph (Figure 4). In this response graph, the highest value of the level for each factor represents the strongest effect. Therefore, optimal parameters were selected based on the highest response values in Figure 4, which were A3 (20% djulis sourdough), B1 (0% addition of hulled djulis), C1 (8% unsalted butter), D2 (80% WF + 20% HGF), and E2 (10% honey). The suggested condition (A3B1C1D2E2) is a generated optimal factor-level combination, which is not among the 16 experimental trials that are listed in Table 2. Although the present study mainly focused on the technological, physical, and sensory properties of the newly developed djulis sourdough bread, it should be noted that the new product developed in the present study can possess unique nutritional values considering previous reports about the bioactive effects of djulis [7]. Further investigation about the nutritional profile, bioactive effects, and potential healthcare applications of this product can be investigated in future studies. Similarly, studies regarding the properties of sourdough bread incorporated with other strains of djulis can be suggested as researchers showed that bread ingredients (e.g., wheat flour) originated from different locations possess various bioactive compounds that can affect the nutritional characteristics of the final product, which might be the case for djulis sourdough bread [30].

#### 3.5.2. ANOVA Analysis

The results of ANOVAs indicate the degrees of influence over multiple quality characteristics (Table 7). As GRA provides a comprehensive analysis of the various characteristics, in the way of balanced consideration of the point of view of consumers, the influences of conflicting factors had already been weakened during the analysis process. Table 7 shows that all five factors (addition/non-addition of djulis sourdough, addition/non-addition of hulled djulis, butter/oil type, WF + HGF, and honey) significantly influenced the quality characteristics of the naturally leavened sourdough bread developed in the present study.

## 4. Conclusions

The present study demonstrated that a combination of Taguchi and grey relational analysis, i.e., a Taguchi–GRA approach, could be employed to investigate the effects of processing parameters on the quality of djulis sourdough bread and to identify the optimal settings for manufacturing new bakery products when multiple characteristics are involved. Such multiple characteristics of bread (e.g., aroma, color, and texture) are important for consumers. At this moment, it seems that no systematic approach has been implanted in the bakery industry that can consider multiple objectives at a time for developing new products. Therefore, the Taguchi–GRA approach, which was explored in this study, could be a prospective optimization technique that can be implemented in the bakery and other sectors of the food industry. The novel Taguchi–GRA approach introduced in this study could provide a reference of the basis for the enhancement of consumer-oriented products in the bakery industry. Furthermore, it was depicted that, sometimes, sensory evaluation could not be the only decisive approach to determine the optimal bakery products. Therefore, a combination of instrumental and sensory analysis can provide realistic data to develop a product with optimal quality parameters. Further studies in the nutritional aspects of such an innovative product can be suggested for future studies.

## Figures and Tables

**Table 1:** Parameter design factors and levels *.

Factors/Level	1	2	3	4
A. Djulis sourdough	0%	10%	20%	30%
B. Hulled djulis	0%	0.5%	1%	1.5%
C. Butter/oil (8%)	Unsalted butter	Camellia oil	Lard	Olive oil
D. WF + HGF **	WF 100%	WF 80% + HGF 20%	WF 60% + HGF 40%	WF 40% + HGF 60%
E. Honey	8%	10%	12%	14%

* The common ingredients were 3.5% fresh yeast, 54% distilled water, 12% egg, 2% salt, and 4% milk powder. ** WF: wheat flour; HGF: high-gluten flour.

**Table 2:** L_16_ (4^5^) orthogonal array and factor/level assignments *.

Factor/Level L_16_ (4^5^)	A	B	C	D	E
1	1	1	1	1	1
2	1	2	2	2	2
3	1	3	3	3	3
4	1	4	4	4	4
5	2	1	2	3	4
6	2	2	1	4	3
7	2	3	4	1	2
8	2	4	3	2	1
9	3	1	3	4	2
10	3	2	4	3	1
11	3	3	1	2	4
12	3	4	2	1	3
13	4	1	4	2	3
14	4	2	3	1	4
15	4	3	2	4	1
16	4	4	1	3	2

* A: djulis sourdough; B: hulled djulis; C: butter/oil (8%); D: wheat flour + high-gluten flour; E: honey.

**Table 3:** Raw data of 14 attributes of 16 trials based on an orthogonal array L_16_(4^5^) (*n* = 3).

Experiment Results														
		Sensory Evaluation data					Texture Analysis data					Color Values		
TN *	Appearance	Aroma	Bitterness	Sourness	Chewiness	Overall Acceptance	Gumminess (kgf)	Chewiness (kgf × mm)	Brittleness (kgf)	Springiness (mm)	Cohesiveness (Ratio)	L*	a*	b*
1	5.37 ± 1.09 **	5.52 ± 0.91	3.97 ± 0.97	3.08 ± 1.16	3.92 ± 1.83	5.85 ± 0.72	0.10 ± 0.01	4.62 ± 0.16	2.13 ± 0.23	5.28 ± 0.11	0.73 ± 0.00	67.42 ± 0.52	-0.03 ± 0.03	14.88 ± 0.15
2	5.83 ± 1.43	2.07 ± 0.76	3.17 ± 1.18	2.83 ± 1.11	3.40 ± 1.62	4.85 ± 0.98	0.16 ± 0.03	4.26 ± 0.15	1.92 ± 0.19	5.48 ± 0.08	0.61 ± 0.01	54.78 ± 0.13	0.80 ± 0.09	14.14 ± 0.27
3	5.77 ± 1.11	4.35 ± 1.10	3.10 ± 0.96	3.10 ± 1.34	3.67 ± 1.79	5.28 ± 1.20	0.09 ± 0.04	3.44 ± 0.43	1.98 ± 0.12	5.19 ± 0.09	0.72 ± 0.01	64.72 ± 0.36	0.13 ± 0.16	14.61 ± 0.52
4	5.40 ± 1.36	4.43 ± 1.04	3.17 ± 0.86	3.15 ± 1.40	3.37 ± 1.76	4.37 ± 1.36	0.06 ± 0.03	3.30 ± 0.19	1.96 ± 0.14	4.92 ± 0.06	0.74 ± 0.06	59.62 ± 0.46	-0.44 ± 0.03	12.15 ± 0.27
5	5.68 ± 1.23	2.17 ± 0.67	3.32 ± 1.16	3.45 ± 0.89	4.25 ± 1.29	3.17 ± 1.40	0.10 ± 0.03	3.94 ± 0.67	1.55 ± 0.26	5.54 ± 0.44	0.82 ± 0.12	52.59 ± 0.34	0.15 ± 0.01	13.22 ± 0.35
6	5.78 ± 1.03	5.08 ± 1.15	3.77 ± 1.12	3.70 ± 1.09	4.45 ± 1.23	3.98 ± 1.48	0.10 ± 0.02	3.99 ± 0.82	1.56 ± 0.28	5.56 ± 0.36	0.81 ± 0.10	50.77 ± 0.22	0.89 ± 0.02	14.52 ± 0.37
7	5.20 ± 1.35	4.58 ± 1.28	3.90 ± 1.20	3.95 ± 1.04	4.48 ± 1.34	4.62 ± 1.21	0.07 ± 0.02	3.77 ± 0.35	1.53 ± 0.27	5.58 ± 0.33	0.78 ± 0.06	61.22 ± 0.18	-0.70 ± 0.04	10.68 ± 0.06
8	5.47 ± 1.37	3.97 ± 1.29	3.85 ± 1.01	3.80 ± 1.07	4.60 ± 1.13	3.88 ± 1.13	0.07 ± 0.03	3.75 ± 0.18	1.54 ± 0.26	4.94 ± 0.18	0.79 ± 0.14	64.34 ± 0.13	-0.42 ± 0.06	11.51 ± 0.22
9	6.97 ± 0.16	6.17 ± 0.77	5.47 ± 1.23	5.58 ± 1.38	6.97 ± 0.14	6.28 ± 0.78	0.19 ± 0.05	4.94 ± 0.30	1.67 ± 0.08	5.64 ± 0.57	0.97 ± 0.01	61.31 ± 0.78	-0.55 ± 0.03	12.10 ± 0.19
10	5.57 ± 1.31	4.55 ± 1.30	4.30 ± 1.05	4.25 ± 0.92	4.85 ± 1.16	5.13 ± 1.30	0.08 ± 0.03	3.16 ± 0.54	1.67 ± 0.26	5.51 ± 0.35	0.79 ± 0.04	67.00 ± 0.17	-0.62 ± 0.08	13.20 ± 0.19
11	5.85 ± 1.17	5.45 ± 0.90	5.00 ± 0.91	5.23 ± 0.75	6.83 ± 0.39	5.95 ± 0.87	0.19 ± 0.03	3.42 ± 0.42	1.67 ± 0.33	5.26 ± 0.12	0.79 ± 0.11	67.92 ± 0.40	-0.32 ± 0.04	12.61 ± 0.27
12	5.98 ± 0.98	2.75 ± 1.13	3.95 ± 1.48	4.15 ± 0.99	4.93 ± 1.15	5.08 ± 1.64	0.08 ± 0.01	3.02 ± 0.50	1.66 ± 0.23	5.30 ± 0.57	0.79 ± 0.04	62.02 ± 0.15	0.07 ± 0.02	10.45 ± 0.06
13	5.03 ± 3.64	4.68 ± 1.14	5.33 ± 1.05	5.05 ± 1.07	6.10 ± 0.67	5.92 ± 0.95	0.06 ± 0.03	3.88 ± 0.32	1.78 ± 0.36	5.50 ± 0.55	0.79 ± 0.04	72.10 ± 0.25	-0.70 ± 0.06	12.75 ± 0.04
14	5.52 ± 1.26	4.62 ± 1.03	3.90 ± 1.18	4.28 ± 1.02	5.08 ± 1.06	4.95 ± 1.22	0.08 ± 0.03	3.34 ± 0.65	1.79 ± 0.08	5.63 ± 0.65	0.79 ± 0.04	65.92 ± 0.34	-0.49 ± 0.05	13.01 ± 0.61
15	5.17 ± 1.60	1.95 ± 0.78	3.27 ± 1.06	3.68 ± 1.19	3.92 ± 1.21	2.52 ± 0.94	0.09 ± 0.04	3.56 ± 0.90	1.80 ± 0.15	5.38 ± 0.11	0.89 ± 0.09	68.55 ± 0.24	-0.91 ± 0.03	9.87 ± 0.14
16	5.37 ± 1.42	4.88 ± 0.94	3.57 ± 1.02	3.83 ± 1.04	4.90 ± 1.03	4.20 ± 1.47	0.16 ± 0.04	4.99 ± 0.98	1.83 ± 0.23	5.53 ± 0.02	0.79 ± 0.11	66.55 ± 0.13	-0.54 ± 0.02	12.76 ± 0.34

* TN: trial number; L*: lightness; a*: green–red coordinate; b*: blue–yellow coordinate; kgf: kilogram-force. ** The results represent the average value of three replications (*n* = 3) followed by standard deviation (SD), i.e., mean ± SD. For sensory evaluation data, *n* = 3 means that 60 panelists repeated the sensory tests on 3 different days.

**Table 4:** Signal-to-noise ratio response table.

S/N Ration														
	Sensory Evaluation						Texture Analysis					Color Values		
Trial No.	Appearance	Aroma	Bitterness	Sourness	Chewiness	OA	Gumminess (kgf *)	Chewiness (kgf × mm)	Brittleness (kgf)	Springiness (mm)	Cohesiveness (Ratio)	L*	a*	b*
1	14.60 **	14.84	11.98	9.77	11.87	15.34	−20.00	13.29	6.57	14.45	−2.73	36.58	−30.46	23.45
2	15.31	6.32	10.02	9.04	10.63	13.71	−15.92	12.59	5.67	14.78	−4.29	34.77	−1.94	23.01
3	15.22	12.77	9.83	9.83	11.29	14.45	−20.92	10.73	5.93	14.30	−2.85	36.22	−17.72	23.29
4	14.65	12.93	10.02	9.97	10.55	12.81	−24.44	10.37	5.85	13.84	−2.62	35.51	−7.13	21.69
5	15.09	6.73	10.42	10.76	12.57	10.02	−20.00	11.91	3.81	14.87	−1.72	34.42	−16.48	22.42
6	15.24	14.12	11.53	11.36	12.97	12.00	−20.00	12.02	3.86	14.90	−1.83	34.11	−1.01	23.24
7	14.32	13.22	11.82	11.93	13.03	13.29	−23.10	11.53	3.69	14.93	−2.16	35.74	−3.10	20.57
8	14.76	11.98	11.71	11.60	13.26	11.78	−23.10	11.48	3.75	13.87	−2.05	36.17	−7.54	21.22
9	16.86	15.81	14.76	14.93	16.86	15.96	−14.42	13.87	4.45	15.03	−0.26	35.75	−5.19	21.66
10	14.92	13.16	12.67	12.57	13.71	14.20	−21.94	9.99	4.45	14.82	−2.05	36.52	−4.15	22.41
11	15.34	14.73	13.98	14.37	16.69	15.49	−14.42	10.68	4.45	14.42	−2.05	36.64	−9.90	22.01
12	15.53	8.79	11.93	12.36	13.86	14.12	−21.94	9.60	4.40	14.49	−2.05	35.85	−23.10	20.38
13	14.03	13.40	14.53	14.07	15.71	15.45	−24.44	11.78	5.01	14.81	−2.05	37.16	−3.10	22.11
14	14.84	13.29	11.82	12.63	14.12	13.89	−21.94	10.47	5.06	15.01	−2.05	36.38	−6.20	22.29
15	14.27	5.80	10.29	11.32	11.87	8.03	−20.92	11.03	5.11	14.62	−1.01	36.72	−0.82	19.89
16	14.60	13.77	11.05	11.66	13.80	12.46	−15.92	13.96	5.25	14.85	−2.05	36.46	−5.35	22.12

* kgf: kilogram-force; OA: overall acceptance; L*: lightness; a*: green–red coordinate; b*: blue–yellow coordinate. ** The results represent the average value of three replications (*n* = 3).

**Table 5:** Data preprocessing for each performance characteristic (*n* = 3).

Data Pretreatment														
	Sensory Evaluation						Texture Analysis					Color Values		
Trial No.	Appearance	Aroma	Bitterness	Sourness	Chewiness	OA	Gumminess (kgf)	Chewiness (kgf × mm)	Hardness (kgf)	Springiness (mm)	Cohesiveness (ratio)	L*	a*	b*
1	0.20	0.90	0.44	0.12	0.21	0.92	0.44	0.85	1.00	0.52	0.39	0.81	0.00	1.00
2	0.45	0.05	0.04	0.00	0.01	0.72	0.85	0.69	0.69	0.79	0.00	0.22	0.96	0.88
3	0.42	0.70	0.00	0.13	0.12	0.81	0.35	0.26	0.78	0.39	0.36	0.69	0.43	0.96
4	0.22	0.71	0.04	0.16	0.00	0.60	0.00	0.18	0.75	0.00	0.42	0.46	0.79	0.51
5	0.37	0.09	0.12	0.29	0.32	0.25	0.44	0.53	0.04	0.87	0.64	0.10	0.47	0.71
6	0.43	0.83	0.34	0.39	0.38	0.50	0.44	0.55	0.06	0.90	0.61	0.00	0.99	0.94
7	0.10	0.74	0.40	0.49	0.39	0.66	0.13	0.44	0.00	0.92	0.53	0.53	0.92	0.19
8	0.26	0.62	0.38	0.43	0.43	0.47	0.13	0.43	0.02	0.03	0.56	0.68	0.77	0.37
9	1.00	1.00	1.00	1.00	1.00	1.00	1.00	0.98	0.26	1.00	1.00	0.54	0.85	0.50
10	0.31	0.74	0.58	0.60	0.50	0.78	0.25	0.09	0.26	0.83	0.56	0.79	0.89	0.71
11	0.46	0.89	0.84	0.90	0.97	0.94	1.00	0.25	0.26	0.49	0.56	0.83	0.69	0.60
12	0.53	0.30	0.43	0.56	0.52	0.77	0.25	0.00	0.25	0.54	0.56	0.57	0.25	0.14
13	0.00	0.76	0.95	0.85	0.82	0.94	0.00	0.50	0.46	0.82	0.56	1.00	0.92	0.62
14	0.28	0.75	0.40	0.61	0.56	0.74	0.25	0.20	0.47	0.99	0.56	0.74	0.82	0.67
15	0.08	0.00	0.09	0.39	0.21	0.00	0.35	0.33	0.49	0.65	0.81	0.86	1.00	0.00
16	0.20	0.80	0.25	0.45	0.52	0.56	0.85	1.00	0.54	0.86	0.56	0.77	0.85	0.63

kgf: kilogram-force; OA: overall acceptance; L*: lightness; a*: green–red coordinate; b*: blue–yellow coordinate.

**Table 6:** Grey relational analysis (GRA) calculation model (ζ = 0.5).

Trial No. *	Appearance	Aroma	Bitterness	Sourness	Chewiness	OA	Gumminess	Chewiness	Hardness	Springiness	Cohesiveness	L*	a*	b*	Grey Relational Grade
Weighting	1/16	1/16	1/16	1/16	1/8	1/8	1/20	1/20	1/20	1/20	1/20	1/12	1/12	1/12	
Reference Sequence	1	1	1	1	1	1	1	1	1	1	1	1	1	1	
1	0.38	0.84	0.47	0.36	0.39	0.87	0.47	0.77	1.00	0.51	0.45	0.72	0.33	1.00	0.6163
2	0.48	0.35	0.34	0.33	0.34	0.64	0.77	0.61	0.61	0.70	0.33	0.39	0.93	0.80	0.5439
3	0.46	0.62	0.33	0.37	0.36	0.72	0.44	0.40	0.69	0.45	0.44	0.62	0.47	0.92	0.5354
4	0.39	0.63	0.34	0.37	0.33	0.56	0.33	0.38	0.67	0.33	0.46	0.48	0.70	0.50	0.4690
5	0.44	0.36	0.36	0.41	0.42	0.40	0.47	0.52	0.34	0.79	0.58	0.36	0.49	0.63	0.4597
6	0.47	0.75	0.43	0.45	0.45	0.50	0.47	0.53	0.35	0.83	0.56	0.33	0.99	0.89	0.5710
7	0.36	0.66	0.46	0.50	0.45	0.60	0.37	0.47	0.33	0.86	0.52	0.52	0.87	0.38	0.5290
8	0.40	0.57	0.45	0.47	0.47	0.49	0.37	0.47	0.34	0.34	0.53	0.61	0.69	0.44	0.4839
9	1.00	1.00	1.00	1.00	1.00	1.00	1.00	0.96	0.40	1.00	1.00	0.52	0.77	0.50	0.8675
10	0.42	0.65	0.54	0.55	0.50	0.69	0.40	0.35	0.40	0.75	0.53	0.71	0.82	0.63	0.5861
11	0.48	0.82	0.76	0.84	0.95	0.89	1.00	0.40	0.40	0.49	0.53	0.75	0.62	0.55	0.7131
12	0.52	0.42	0.47	0.53	0.51	0.68	0.40	0.33	0.40	0.52	0.53	0.54	0.40	0.37	0.4881
13	0.33	0.68	0.92	0.77	0.73	0.89	0.33	0.50	0.48	0.73	0.53	1.00	0.87	0.57	0.7026
14	0.41	0.67	0.46	0.56	0.53	0.66	0.40	0.38	0.49	0.97	0.53	0.66	0.73	0.60	0.5855
15	0.35	0.33	0.36	0.45	0.39	0.33	0.44	0.43	0.50	0.59	0.73	0.78	1.00	0.33	0.4930
16	0.38	0.71	0.40	0.47	0.51	0.53	0.77	1.00	0.52	0.78	0.53	0.69	0.77	0.57	0.6016

* OA: overall acceptance; L*: lightness; a*: green–red coordinate; b*: blue–yellow coordinate.

**Table 7:** The summary of ANOVA results.

	SS *	DF **	Variance	F-Ratio	Confidence (%)	Significant
A	0.1622	3	0.0541	116.98	100.00%	***
B	0.1399	3	0.0466	100.91	100.00%	***
C	0.1280	3	0.0427	92.32	100.00%	***
D	0.0379	3	0.0126	27.33	100.00%	***
E	0.0584	3	0.0195	42.14	100.00%	***
Error	0.0148	32	0.0005			
Total	0.5411	47				

* SS: sum of squares. ** DF: degrees of freedom. *** *p* < 0.001.

## Supplementary Materials

The following are available online at https://www.mdpi.com/2304-8158/9/9/1149/s1, Figure S1: The raw sensory data from questionnaires.
